# Prospects for the application of pathological response rate in neoadjuvant therapy for gastric cancer

**DOI:** 10.3389/fonc.2025.1528529

**Published:** 2025-04-11

**Authors:** Zicheng Bao, Nan Jia, Zhidong Zhang, Chenyu Hou, Bin Yao, Yong Li

**Affiliations:** The Third Department of Surgery, The Fourth Hospital of Hebei Medical University, Shijiazhuang, China

**Keywords:** gastric cancer, perioperative period, chemotherapy, immunotherapy, pathological remission, prognosis

## Abstract

With the annual increase in the incidence and mortality rates of gastric cancer, it has gradually become one of the significant threats to human health. Approximately 90% of gastric cancer patients are diagnosed with adenocarcinoma. Although the 5-year survival rate for early-stage gastric cancer can exceed 90%, due to its concealed symptoms, less than half of the patients are eligible for radical surgical treatment upon diagnosis. For gastric cancer patients receiving palliative treatment, the current expected survival time is only about one year. In China, the majority of gastric cancer patients, accounting for about 80% of the total, are in the locally advanced stage. For these patients, radical surgery remains the primary treatment option; however, surgery alone is often inadequate in controlling tumor progression. In the pivotal MAGIC study, the recurrence rate was as high as 75%, and similar results were obtained in the French ACCORD07-FFCD9703 study. Numerous clinical trials are currently exploring preoperative neoadjuvant therapy for patients with locally advanced gastric cancer. Data indicates that preoperative neoadjuvant therapy can not only reduce the size of the local tumor but also shrink surrounding lymph nodes, thereby downstaging the tumor and improving the R0 resection rate. Additionally, it can decrease tumor cell activity and eliminate potential micrometastases. The emergence of various immunotherapies has ushered in a new era for neoadjuvant treatment options for gastric cancer.

## 1 Introduction

With nearly 1 million new cases each year, gastric cancer is not only the fifth most common cancer globally but also the fourth leading cause of cancer-related deaths (1). The number of deaths from gastric cancer accounts for approximately 7.7% of the total cancer deaths (2). Adenocarcinoma is the most predominant pathological histotype in gastric cancer, accounting for about 90% of all gastric malignancies (3). The overall case-fatality rate of gastric cancer is approximately 75% (4). The 5-year survival rate for patients with advanced gastric cancer is only about 10% (5). In recent years, the emergence of immunotherapy has transformed previous treatment paradigms, with an increasing number of immune-based drugs being utilized in the adjuvant therapy for gastric cancer patients. Immunotherapy has demonstrated its advantages in a series of clinical trials for neoadjuvant therapy in gastric cancer. However, these trials have predominantly focused on Overall Survival (OS), Progression-Free Survival (PFS), and Disease-Free Survival (DFS) as their primary endpoints. Yet, such studies are often constrained by long time spans and high investment costs. Consequently, some trials compare the efficacy of drugs by assessing the pathological complete response (PCR) rate in surgical specimens after neoadjuvant therapy. Nonetheless, the number of patients achieving PCR is relatively small, making PCR rate alone a limited metric. This has led to the consideration of Major Pathological Response (MPR) as a new indicator for evaluating the efficacy of neoadjuvant therapy and predicting prognosis. This article aims to provide an overview of the research progress on MPR and its application in neoadjuvant therapy for gastric cancer.

## 2 Overview of immuno-neoadjuvant therapy for gastric cancer

### 2.1 Immune checkpoints and the mechanism of action of immune checkpoint inhibitors

The immune system of a healthy individual maintains a dynamic equilibrium. A decrease in immune response can lead to an increased risk of tumor development, while similarly, an enhanced immune response can result in the onset of autoimmune diseases (6). Immunomodulatory approaches primarily involve the use of monoclonal antibodies or recombinant fusion proteins that target cell surface signaling molecules on immune cells to drive the immune response in the desired direction. The immune system has the ability to recognize tumor antigens present in the body, playing an extremely important role in tumor prevention.

Immune checkpoint inhibitors (ICIs) can effectively block immunosuppressive cells and activate the body’s own anti-tumor immune function by blocking relevant signaling pathways and reactivating T-cells, thereby enhancing the anti-tumor effect of the immune system (7). Programmed Cell Death Protein 1 (PD-1) is an immunosuppressive receptor primarily expressed on activated T-cells, B-cells, natural killer cells, monocytes, and some tumor cells (8, 9). Programmed Cell Death Protein 1 Ligand (PD-L1) is the ligand for PD-1, mainly expressed on the surface of parenchymal cells and tumor cells (10). When PD-1 binds to PD-L1, it can inhibit the immune response of T-cells by binding to downstream signaling molecules (11). Immunotherapy targeting PD-1/PD-L1 signaling is not simply about enhancing the function of immune cells in tumors, but rather about normalizing the immune system (12).

However, studies have also shown that as tumors develop, the combination of PD-1 and PD-L1 inhibits the host’s anti-tumor immunity, leading to tumor immune escape: 1. Inhibiting the activation of tumor-infiltrating lymphocytes (TILs) and inducing their apoptosis; 2. Suppressing the production of granzyme and perforin by cytotoxic T lymphocytes (CTLs); 3. Reducing the secretion of inflammatory cytokines and promoting the secretion of the immunosuppressive cytokine IL-10; 4. Promoting tumor cell epithelialization, metastasis, and infiltration (13–15). In terms of cellular immunity, T-cells specifically recognize antigens and activate immune responses, inhibiting immune checkpoints on tumor cells and playing a crucial role in lifting T-cell immunosuppression. Immunotherapy has become an effective approach in anti-tumor treatment (16). The latest results of antibody therapy targeting PD-1 and PD-L1 in various advanced gastric cancers indicate that immunotherapy has matured (17).

### 2.2 Progress in immunotherapy for advanced gastric cancer

In recent years, with the research on immune drugs such as immune checkpoint inhibitors, immunotherapy has become one of the key research areas in the overall treatment of gastric cancer (18). The existing immune drugs include Sintilimab, Toripalimab, Pembrolizumab, Nivolumab, etc. In 2023, China’s ORIENT-16 trial enrolled a total of 650 patients with advanced gastric cancer or gastroesophageal junction adenocarcinoma, who were randomly assigned to the Sintilimab treatment group and the placebo group. The placebo group received Sintilimab combined with Capecitabine and Oxaliplatin (XELOX regimen), while the control group received placebo combined with the XELOX regimen. The results showed that the Sintilimab group had a significantly improved overall survival (OS) compared to the placebo group (15.2 months *vs*. 12.3 months). Among patients in the Sintilimab group with a Combined Positive Score (CPS) of 5 or higher, the OS reached 18.4 months. The objective response rate (ORR) in the Sintilimab group was also significantly higher than that in the placebo group (63.6% *vs*. 49.4%) (19). Trials such as RATIONALE 305 have also demonstrated the value of immunotherapy in first-line treatment for advanced gastric cancer, significantly improving patients’ survival benefits (20). With the conduct of multiple clinical trials for gastric cancer, programmed death-1 (PD-1)/programmed death ligand-1 (PD-L1) monoclonal antibodies have been approved for first-line treatment of advanced gastric cancer, and the application of immunotherapy in the perioperative period of gastric cancer is also being explored. A multicenter, randomized, open-label phase 3 clinical trial (CheckMate 649) in 2021 enrolled 1,581 patients with advanced gastric cancer or gastroesophageal junction or esophageal adenocarcinoma. The patients were randomly assigned to the Nivolumab plus chemotherapy group and the chemotherapy-alone group. The results showed that the Nivolumab plus chemotherapy group had a superior overall survival compared to the chemotherapy-alone group (13.1 months *vs*. 11.1 months) and also significantly improved patients’ progression-free survival (21). And the latest 3-year follow-up results of this trial showed that Nivolumab combined with chemotherapy significantly improved patients’ OS (21% *vs*. 10%), PFS (13% vs. 8%), and ORR (60% *vs*. 45%) compared to chemotherapy alone (22). In enrolled patients with a CPS of ≥5, Nivolumab combined with chemotherapy reduced the risk of recurrence (23).

PD-L1 expression and microsatellite instability (MSI) status are typically assessed during the initial diagnostic biopsy. For example, combined positive score (CPS) ≥5 is a widely accepted threshold for PD-L1 positivity, which guides the selection of immune checkpoint inhibitors. HER2 status is also routinely evaluated via immunohistochemistry (IHC) or fluorescence *in situ* hybridization (FISH) at this stage.

### 2.3 The development process of neoadjuvant immunotherapy for gastric cancer

Although the treatment of gastric cancer has developed rapidly in recent years, surgery remains the mainstay of treatment for locally advanced gastric cancer. Although most patients can achieve R0 surgical resection, the overall 5-year survival rate is not high. Moreover, with the increasing incidence of autoimmune gastritis, imbalance of gastric flora, and the use of antibiotics and acid suppressants, the incidence of gastric cancer continues to rise (2). For patients with resectable gastric cancer, most domestic and international guidelines recommend a treatment regimen that combines surgery with postoperative adjuvant chemotherapy, radiotherapy, targeted therapy, and immunotherapy (18). Despite undergoing radical surgery, patients with locally advanced gastric cancer still face a high risk of postoperative recurrence. Compared with traditional surgery combined with postoperative adjuvant chemotherapy, neoadjuvant chemotherapy combined with surgical resection has improved the OS of postoperative gastric cancer patients by 13% (24). In recent years, the treatment strategy for patients with locally advanced gastric cancer is shifting from the traditional approach of surgery followed by postoperative adjuvant therapy to preoperative neoadjuvant therapy combined with surgical treatment. Radical surgical resection remains the preferred treatment option for gastric cancer patients to achieve cure. Locally advanced gastric cancer mainly refers to gastric cancer before stage IV, where the tumor is locally advanced but no distant metastasis is found. It is difficult to achieve radical surgical resection for patients with distant metastasis. The main purpose of preoperative neoadjuvant therapy is to expand the target population and increase the possibility of radical surgical resection for more gastric cancer patients.

PD-1/PD-L1 inhibitors used alone or in combination as first-line therapy have demonstrated good long-term survival rates in various advanced cancers, including melanoma, lung cancer, gastric cancer, and others (25). As early as 2020, the success of the Checkmate-649 trial led to the PD-1 inhibitor finally entering the first-line treatment for advanced gastric cancer in China. The survival duration for advanced gastric cancer was improved from the previous 10.2 months to 14.3 months, and the median survival time was also increased from 5.6 months to 12.2 months. With acceptable safety, it effectively and significantly improved patients’ OS and PFS, and officially changed the treatment dilemma for Her-2 negative patients in China (26).

### 2.4 Research progress in neoadjuvant immunotherapy for gastric cancer

ICI (Immune Checkpoint Inhibitors) block the signaling pathway between T lymphocytes and antigen-presenting cells by binding to tissue immune checkpoint molecules and their ligands, thereby enhancing immune cell activity, breaking the immune tolerance of tumor cells, and activating the body’s cellular immunity to eliminate tumor cells, thus inhibiting the growth and proliferation of tumors (27). Similar to traditional neoadjuvant chemotherapy, the antitumor effect of neoadjuvant immunotherapy can also shrink tumor lesions, achieve downstaging, and improve the R0 resection rate. At the same time, the immune activation effect generated by patients after receiving immunotherapy can also eliminate micrometastases in the tumor (28). It has been reported that neoadjuvant immunotherapy can not only enhance the expression of PD-L1 within tumor tissues but also promote the infiltration of immune cells into the tumor tissues (29).

Existing research results also indicate that neoadjuvant immunotherapy is more rational compared to traditional neoadjuvant chemotherapy regimens. Tumor tissues contain many immunotherapy-related targets, including antibodies against cytotoxic T lymphocyte-associated antigen 4 (CTLA4) that induce antitumor immunity, PD-1 that restricts T-cell effector function within tissues, and PD-L1 that blocks antitumor immune responses in the tumor microenvironment. Due to the presence of these targets, neoadjuvant immunotherapy can activate infiltrating lymphocytes in the tumor microenvironment through the abundance of tumor-associated antigens, thereby generating a more intense and durable antitumor response (30). Additionally, neoadjuvant immunotherapy can induce cellular and humoral immunity in the body, resulting in long-term immune memory. This immune memory may have the function of preventing tumor recurrence, which may also explain why traditional neoadjuvant chemotherapy patients fail to produce long-lasting antitumor effects through immune mediation after undergoing radical surgical resection of the primary tumor (31). Furthermore, when applying ICI, the abundant tumor-associated antigens infiltrating within the tumor tissue can be used for cross-linking reactions. Immunotherapy can reactivate T lymphocytes, enabling them to circulate from the primary tumor site, thereby addressing the issue of tumor micrometastasis (32, 33).

Numerous clinical trial results have demonstrated the superiority and scientific nature of neoadjuvant immunotherapy, showing significant advantages in local tumor downstaging, improving R0 resection rates, and reducing tumor micrometastasis. However, due to the relatively late start of immunotherapy, many clinical research results on neoadjuvant immunotherapy have not yet been published. Existing results indicate that neoadjuvant immunotherapy can significantly increase the PCR (pathological complete response) rate in patients, but the number of patients achieving pathological complete remission is still small. The PCR rate for melanoma is approximately 19-43% (34, 35). The PCR rate for lung cancer patients is only 5-15% (36, 37). Long-term follow-up chemotherapy studies have shown that MPR (major pathological response) can predict long-term survival in patients, and this is also true for patients receiving immunotherapy (34, 38). Therefore, using MPR as a surrogate endpoint for research is a viable option.

In multidisciplinary tumor boards, imaging modalities such as contrast-enhanced CT scans are critical for evaluating tumor invasion depth (T stage), lymph node involvement (N stage), and potential peritoneal carcinomatosis. Exploratory laparoscopy with peritoneal cancer index (PCI) scoring is recommended for suspected peritoneal metastasis. For patients with high PCI scores, pressurized intraperitoneal aerosol chemotherapy (PIPAC) may be integrated into neoadjuvant regimens to achieve tumor downstaging and conversion to resectability.

## 3 Pathological response rate

### 3.1 Pathological response pattern of gastric cancer after immunotherapy

After the application of immune checkpoint inhibitors (ICIs) in patients with gastric cancer, the tumors exhibit varying degrees of regression. However, there is currently no clear definition for the changes in pathologic response following neoadjuvant therapy. The immune-related pathologic response (irPR) observed in tumor specimens after immunotherapy primarily exhibits features of immune activation, cell death, tissue repair, and the presence of a fibrous scar. Immune activation is characterized by lymphatic infiltration, tertiary lymphoid structures, and plasma cells observed under a light microscope. Cell death is marked by the presence of foamy macrophages and cholesterol clefts. Tissue repair manifests as proliferative fibrosis and neovascularization, while the fibrous scar often remains at the periphery of the tumor (39).

### 3.2 The criteria for assessing pathologic response

The main histological features of local lesions after neoadjuvant therapy include: (1) residual tumor; (2) necrosis; and (3) stromal tissue (including inflammatory and fibrotic tissue). In 2017, the College of American Pathologists (CAP) proposed a pathological assessment model for resected tumor tissue after neoadjuvant therapy, which records the presence of residual viable tumor greater than or equal to 10%. Subsequently, the Royal College of Pathologists in the United Kingdom recommended the same threshold (40). The efficacy of neoadjuvant therapy is assessed based on the amount of residual tumor cells in the local tumor after treatment. Currently, the Tumor Regression Grade (TRG) is commonly used in clinical practice to evaluate the pathological regression of gastric cancer after neoadjuvant therapy. This assessment scheme categorizes the degree of tumor residue in postoperative pathological specimens into the following four grades: Grade 0: Complete regression, no residual tumor cells (including in lymph nodes); Grade 1: Moderate regression, only single or small foci of residual cells; Grade 2: Minimal regression, tumor residue but less than fibrotic stroma; Grade 3: No regression, extensive tumor residue with no or minimal tumor cell necrosis (41, 42). There is also a definition of TRG grading based on the percentage of viable tumor cells in the specimen, where TRG 0 = 0%, TRG 1 = 1-2%, TRG 2 = 3-50%, and TRG 3 ≥ 50%. According to this definition, patients with TRG 0 and TRG 1 can be classified as having a major pathological response (MPR), while patients with TRG 3 are classified as non-MPR. As for patients with TRG 2, further assessment is required to make a determination (43). Numerous evidence suggests that pathological response is closely related to survival, and it has been supported by regulatory agencies such as the U.S. Food and Drug Administration (FDA) as an important evaluation indicator for accelerated approval of new therapies for breast cancer (44).

### 3.3 Major pathological response and complete pathological response

Currently, the commonly used indicators to evaluate the efficacy of neoadjuvant therapy in clinical practice include MPR and PCR. Complete pathological response (PCR) refers to the absence of any residual tumor cells under the microscope in the tumor bed of the surgically resected specimen after treatment and HE staining. Major pathological response (MPR) refers to the presence of residual tumor cells in the tumor bed that are ≤10% (45). In the past, different countries have used varying thresholds to predict the optimal cutoff value, with Western countries often adopting 10% or 50% as the critical percentage (46, 47). Asian countries, on the other hand, typically use the thresholds of 33% or 67% as defined in the Japanese Classification of Gastric Carcinoma (48). A retrospective study has confirmed that a cutoff value of 10% can effectively predict patient survival (49).

### 3.4 The application of pathological response rate in neoadjuvant chemotherapy for gastric cancer

As early as 1982, there were reports on the use of pathological response rate to evaluate the efficacy of neoadjuvant therapy. After neoadjuvant chemotherapy, histological examination has predictive significance for the survival of patients with a pathological response (50). With the continuous development of chemotherapy drugs and the emergence of different treatment regimens, numerous clinical trials and studies have supported that PCR is related to the prognosis and long-term survival of potentially resectable gastric cancer patients receiving neoadjuvant chemotherapy. However, relatively few patients achieve PCR in clinical practice, so whether MPR can be used as an observational endpoint remains to be discussed.

The British MAGIC trial enrolled a total of 503 patients with locally advanced gastric cancer, who were randomly divided into a surgery-alone group (253 patients) and a perioperative chemotherapy plus surgery group (250 patients). The chemotherapy regimen consisted of 3 cycles of ECF (epirubicin + cisplatin + fluorouracil) administered preoperatively and postoperatively. The results showed that the incidence of postoperative complications was similar between the perioperative chemotherapy group and the surgery group, and the number of deaths within 30 days after surgery was also similar. The perioperative chemotherapy group had a higher rate of radical resection, significantly smaller tumors, and slower progression. With a median follow-up of 4 years, the perioperative chemotherapy group had a higher overall survival rate compared to the surgery group (51). The OEO2 trial by the Medical Research Council (MRC) in the UK enrolled 802 patients with resectable esophageal cancer to compare surgery alone versus preoperative cisplatin + fluorouracil for 2 cycles. Long-term follow-up confirmed that preoperative chemotherapy can improve the overall survival (OS) of patients with resectable esophageal cancer (52). The French FNLCC/FFCD multicenter trial enrolled a total of 224 patients with resectable adenocarcinoma of the lower esophagus, gastroesophageal junction, or stomach, and randomly assigned them to a perioperative chemotherapy group and a surgery-alone group. The chemotherapy regimen was CF (fluorouracil + cisplatin). The results showed that the perioperative chemotherapy group had higher R0 resection rates, disease-free survival (DFS), and overall survival (OS) compared to the surgery-alone group, with a 14% improvement in 5-year survival. However, there was no statistically significant difference in the number of lymph node metastases (53). A clinical trial in Europe enrolled a total of 144 patients with locally advanced gastric cancer or adenocarcinoma of the esophagogastric junction, who were randomly assigned to a surgery-alone group and a neoadjuvant chemotherapy group. The neoadjuvant chemotherapy group received 2 cycles of cisplatin + leucovorin + fluorouracil preoperatively. The results showed that the surgery-alone group had more lymph node metastases than the neoadjuvant chemotherapy group, and the neoadjuvant chemotherapy group had a higher R0 resection rate. The local tumors were often smaller in the neoadjuvant chemotherapy group compared to the surgery-alone group. Five patients achieved a pathological complete response after neoadjuvant chemotherapy. There were 41 patients (58.6%) in the neoadjuvant chemotherapy group who had no lymph node metastases, while only 23 patients (33.8%) in the surgery-alone group had no lymph node metastases. However, postoperative complications were more common in the neoadjuvant chemotherapy group, with 3 patients dying from postoperative complications, while only 1 patient in the surgery-alone group died. There was no statistically significant difference in overall survival (54). A German phase 2/3 clinical trial enrolled 265 patients with resectable gastric or gastroesophageal junction cancer, who were randomly assigned to the FLOT (docetaxel + oxaliplatin + leucovorin + fluorouracil) group (128 patients) and the ECF/ECX (epirubicin + cisplatin + fluorouracil) group (137 patients). Patients in the FLOT group received 3 cycles of preoperative chemotherapy, while those in the ECF/ECX group received 4 cycles. The results showed that the proportion of patients achieving PCR was higher in the FLOT group compared to the ECF/ECX group (55). Although the results of this trial indicate that the perioperative FLOT regimen is effective and feasible, and may potentially serve as a treatment option for locally advanced, resectable gastric or gastroesophageal junction adenocarcinoma, the trial lacks long-term follow-up data, making it uncertain whether FLOT can improve patients’ long-term survival. Previous clinical trials on neoadjuvant chemotherapy were conducted earlier, and while the perioperative chemotherapy group significantly improved the R0 resection rate, there is no clear evidence that perioperative neoadjuvant chemotherapy can improve overall survival or slow disease progression. Different patients respond differently to perioperative neoadjuvant chemotherapy, with varying effects. Separate follow-up was not conducted for the subset of patients who achieved PCR, so it is unclear whether their long-term prognosis is better than that of patients who underwent surgery alone. Studies have shown that administering 8 cycles of FLOT chemotherapy preoperatively resulted in a PCR rate of 18.2%, a lymph node negativity rate of 39.4%, and an OS of 21.3 months, demonstrating certain advantages over direct surgery in reducing tumor micrometastases and prolonging patient prognosis (56).

The MPR (major pathological response) rate has seen a certain degree of improvement in neoadjuvant targeted therapy. In a single-arm phase II trial conducted by Li Song et al.25 potentially resectable patients were enrolled to receive a combination therapy of ICI (immune checkpoint inhibitors) + Apatinib + SOX (oxaliplatin + S-1). A total of 26.3% of the patients achieved MPR (57). Another phase II clinical trial administered a combination therapy of capecitabine, oxaliplatin, and irinotecan to 40 patients with resectable gastric cancer for 4 cycles. As a result, 36 patients successfully completed surgical treatment, and postoperative pathological reports indicated that 36 patients achieved MPR (major pathological response) (58). The results of a phase II clinical trial combining trastuzumab with FLOT reported a PCR rate of 21.4% and an MPR rate of 25%. Another similar phase II clinical trial reported a PCR rate of 35%, and the combination of FLOT with trastuzumab/pertuzumab significantly increased the PCR rate, with a lower lymph node positivity rate, greatly improving tumor micrometastasis (59). In such studies, pathological response rates are rarely used as the primary endpoint. However, it is not difficult to observe that, despite changes in treatment regimens, the MPR rates of neoadjuvant chemotherapy or neoadjuvant targeted therapy are far lower than those of neoadjuvant immunotherapy.

## 4 Application of pathological remission in neoadjuvant immunotherapy for gastric cancer

### 4.1 Neoadjuvant immunotherapy with a single immunotherapeutic agent

In the early stages of the development of neoadjuvant immunotherapy, many related clinical trials began with neoadjuvant monotherapy using a single immunotherapeutic agent. A multicenter, open-label, single-arm phase I clinical trial enrolled 31 patients with resectable gastric cancer, who were administered neoadjuvant immunotherapy with nivolumab alone for 2 cycles. Among them, 30 patients successfully completed surgical treatment, and postoperative pathological reports showed that 5 patients achieved MPR and 1 patient achieved PCR. In this study, it was found that patients who achieved MPR had characteristics of high PD-L1 expression, high microsatellite instability, and/or high tumor mutational burden (60). ATTRACTION-2 is a phase III clinical trial comparing nivolumab to placebo, which enrolled a total of 493 patients. The results showed that the nivolumab group had a significantly higher overall survival (OS) rate compared to the placebo group (1-year: 27.3% *vs* 11.6%; 2-year: 10.6% vs 3.2%), and patients benefited regardless of their tumor’s PD-L1 expression (61).

### 4.2 Neoadjuvant therapy combining immunotherapy with chemotherapy

A multicenter real-world clinical study in China enrolled a total of 585 patients, including 195 in the neoadjuvant immunotherapy group and 390 in the neoadjuvant chemotherapy group. The results showed that the neoadjuvant immunotherapy group had higher rates of pathological complete response (14.36%) and major pathological response (39.49%) compared to the neoadjuvant chemotherapy group (6.41% and 16.15%, respectively). Additionally, neoadjuvant immunotherapy reduced the risk of early recurrence in patients. However, long-term follow-up results for the patients are lacking (62). In 2022, a phase 1 clinical trial enrolled 31 patients with locally resectable gastric adenocarcinoma. Patients were administered a single agent, nivolumab, for 2 cycles preoperatively. During the treatment period, one patient was unable to undergo surgery due to liver metastasis. Of the remaining 30 patients, 27 achieved radical surgery with an R0 resection rate of 90%. Among them, 5 patients (16%) achieved major pathological response (MPR), and 1 patient achieved pathological complete response (PCR). The limitation of this trial is that patients were only administered immunotherapy without the concurrent use of cytotoxic drugs, thus it is unclear whether the efficacy of immunotherapy is superior to that of chemotherapy alone (60). In 2024, a phase 2 clinical trial (NEOSUMMIT-01) was reported, which enrolled 108 patients with resectable gastric cancer or gastroesophageal junction adenocarcinoma at preoperative imaging stages of cT3-4aN+M0. The patients were randomly divided into a toripalimab plus chemotherapy group and a chemotherapy-alone group. The toripalimab plus chemotherapy group received 3 preoperative and 5 postoperative cycles of toripalimab combined with SOX/XELOX regimen, followed by 6 months of toripalimab monotherapy. The chemotherapy-alone group received 3 preoperative and 5 postoperative cycles of SOX/XELOX regimen. The results showed that the R0 resection rates were comparable between the two groups. The toripalimab plus chemotherapy group had a higher proportion of TRG0/1 than the chemotherapy-alone group (44.4% *vs* 20.4%), and a superior PCR rate (22.2% *vs* 7.4%). The incidence of surgical complications was lower in the toripalimab plus chemotherapy group compared to the chemotherapy-alone group (11.8% *vs* 13.5%) (63). Compared to chemotherapy alone, the combination of chemotherapy and toripalimab significantly increased the proportion of patients achieving TRG0/1 and demonstrated manageable safety. A limitation of this trial is that it did not extensively discuss whether patients with TRG2 could achieve pathological response based on postoperative pathology, and postoperative follow-up data for the patients were not reported. KEYNOTE-585 is a multicenter, randomized, placebo-controlled, double-blind phase 3 clinical trial that evaluated perioperative use of pembrolizumab combined with chemotherapy. It enrolled 804 patients with locally advanced, resectable gastric or gastroesophageal junction adenocarcinoma, who were randomly assigned to receive pembrolizumab plus chemotherapy or placebo plus cisplatin-based chemotherapy. Both groups received 3 preoperative and 11 postoperative cycles of treatment. The results showed that the pembrolizumab plus cisplatin-based chemotherapy group had a higher pathological complete response rate than the placebo group (12.9% *vs* 2%), and a significantly longer progression-free survival (44.4 months *vs* 25.3 months). However, there was no significant difference in overall survival between the two groups (60.7 months *vs* 58.0 months). Although this trial demonstrated significant benefits in pathological response, there was no clear benefit in overall survival. Therefore, further exploration is needed to determine whether immunotherapy can be widely used in the neoadjuvant and adjuvant treatment of gastric cancer patients (64). In the PERSIST trial conducted by Tianjin Medical University Cancer Institute & Hospital, all 21 enrolled patients completed 3 preoperative cycles of sintilimab combined with SOX regimen. All 21 patients achieved R0 resection, among which 7 patients (33.3%) achieved pathological complete response (PCR), and 8 patients (38.1%) achieved major pathological response (TRG0-1). The results suggest that administering sintilimab combined with SOX regimen during the perioperative period can achieve high rates of PCR and major pathological response (65). The results of the DANTE trial, presented at the 2020 ASCO Annual Meeting, demonstrated that the combination of atezolizumab with the FLOT regimen improved both the R0 resection rate and the pathological response rate compared to the FLOT regimen alone. Moreover, patients with high PD-L1 expression and MSI-H status benefited even more significantly. However, there was a lack of reporting on patient prognosis (66). A real-world study conducted by the School of Statistics and Mathematics, Huazhong University of Science and Technology, and Wuhan Union Hospital included 119 patients receiving neoadjuvant chemotherapy alone and 50 patients receiving neoadjuvant chemotherapy plus tislelizumab. The results showed no statistically significant difference in the incidence of postoperative complications between the two groups (24.4% *vs* 26%). The R0 resection rate was higher in the neoadjuvant chemotherapy plus tislelizumab group compared to the neoadjuvant chemotherapy alone group (100% *vs* 89.9%), and the pathological complete response rate was also higher (26% *vs* 3.4%). Local tumor regression was more pronounced in the combination group. However, the long-term prognosis of this trial remains to be studied (67) A phase II clinical trial of neoadjuvant sintilimab combined with FLOT reported a high MPR rate of 55.2% and a PCR rate of 17.2%. Patients who achieved PCR demonstrated significant advantages in terms of EFS, OS, and DFS compared to non-PCR patients (68).

While FLOT (docetaxel, oxaliplatin, leucovorin, fluorouracil) is a standard perioperative regimen, DOC (69) (docetaxel, oxaliplatin, capecitabine) offers comparable efficacy with reduced gastrointestinal toxicity due to alternative administration routes. Surgical approaches (open, laparoscopic, or robotic) should be tailored to tumor location and surgeon expertise. D2 (70) lymphadenectomy remains the gold standard, though extended lymph node dissection (e.g., para-aortic nodes) may be feasible in open surgery for selected cases.

## 5 Summary and prospects

In summary, the rapid development of neoadjuvant immunotherapy has gradually emerged as a prominent treatment option for potentially resectable gastric cancer patients. With the gradual deepening of multiple clinical trials, clinicians are also increasingly promoting this treatment mode (71). The MPR rate of postoperative tumor tissue has been proven to be correlated with prognosis and long-term benefits, suggesting its potential as a primary endpoint in neoadjuvant immunotherapy research, which in turn drives drug development. However, there are several controversies: 1. There is no clear and unified international standard for pathological evaluation, making it difficult to ensure the quality of pathological diagnosis, which is a major obstacle to its widespread adoption. 2. There are differences between pathological response rates and radiological evaluations, with RECIST criteria still being the primary standard in clinical practice. How to balance these two is an issue. 3. Further research is needed to determine the consistency between MPR as a routine research focus and PFS, OS, and DFS. 4. The clinical factors influencing MPR are not yet clear, and identifying a more precise population of beneficiaries is also a key issue. Therefore, more relevant studies are needed to comprehensively describe the relationship between MPR and neoadjuvant therapy for gastric cancer, in order to standardize treatment regimens and improve patient prognosis.

Future research should prioritize standardizing pathological assessment protocols to ensure reproducibility. Prospective trials are needed to validate MPR as a surrogate for long-term survival and explore combinatorial strategies. Additionally, integrating liquid biopsies (ctDNA) and radiomics may enable real-time monitoring of treatment response. Clinically, MPR could guide personalized adjuvant therapy, such as de-escalation in responders or intensification in non-responders.
